# Pharmacological Effects of Natural Products: Antioxidant, Anti-Inflammatory and Anticancer Activities

**DOI:** 10.3390/molecules31173012

**Published:** 2026-08-28

**Authors:** Juan Carlos Sepúlveda-Arias, Ramón Enrique Robles-Zepeda

**Affiliations:** 1 Grupo Infección e Inmunidad, Facultad de Ciencias de la Salud, Universidad Tecnológica de Pereira, Pereira 660003, Colombia; 2 Departamento de Ciencias Químico-Biológicas, Universidad de Sonora, Hermosillo 83000, Mexico

Ethnopharmacological knowledge of plant use in treating diseases has facilitated the identification of therapeutic agents against various diseases, including inflammatory diseases and cancer. Approximately 75% of the drugs used for cancer treatment are derived from or inspired by plants [1,2]. In the current paradigm of drug discovery, natural products remain a strategic cornerstone for identifying novel therapeutic leads with high structural diversity. The biological triad of oxidative stress, chronic inflammation, and cancer represents a critical intersection in human pathology, where cancer is considered a chronic inflammatory disease. Persistent oxidative stress also drives cellular transformation and malignant progression. This Special Issue of *Molecules* highlights the importance of natural products and molecules of natural origin in developing anti-inflammatory and anticancer agents.

In this Special Issue, Luzardo-Ocampo et al. report the evaluation of the cytotoxic and proapoptotic effects of Andean berry (*Vaccinium meridionale* Swartz) juice (ABJ) in human SW480 and SW620 colon cancer cell lines, which represent early-stage and metastatic colorectal cancer, respectively. The results of this study reveal a significant stage-specific bioactivity: ABJ markedly reduces the metabolic activity and proliferation, with the most potent effects observed in metastatic SW620 cells, and triggered phase-specific cell cycle arrest, targeting the S phase in SW480 cells and the G2/M transition in SW620 cells. Proteomic and bioinformatics analyses indicate that ABJ promotes extrinsic apoptosis in early-stage SW480 cells, whereas intrinsic apoptosis is the primary pathway modulated in metastatic SW620 cells. In silico analysis was able to predict molecular interactions between apoptosis markers and the phytochemicals present in Andean berry juice, such as chlorogenic acid, caffeic acid, and cyanidin 3-glucoside [Contribution 1].

Pang et al. investigate the ability of Bufalin, a cardiotonic steroid isolated from toad skin, to suppress colorectal liver metastasis. The strategic significance of this work lies in its focus on metabolic vulnerabilities; the study details how Bufalin inhibits de novo fatty acid synthesis by disrupting the PI3K/AKT-mediated SREBP1/FASN signaling axis. By reducing fatty acid synthase (FASN) expression, Bufalin effectively curtails the energy and structural resources required for colorectal cancer progression and secondary colonization in the liver [Contribution 2]. Together, these studies demonstrate the ability of molecules of natural origin to act as multitarget regulators of both apoptotic signaling and metabolic homeostasis in cancer.

Validating pharmacological potential in the modern era relies on a paradigm shift from crude extract therapy to molecularly defined extracts or pure molecules. Advanced analytical techniques, such as GC–MS and LC–ESI–MS/MS, are not only mere descriptive tools but also essential prerequisites for ensuring reproducibility and identifying the precise bioactive molecule within a plant extract. Mhinana et al. provide a rigorous characterization of volatile compounds in *Euryops floribundus* leaves, identifying a chemotype rich in monoterpenes (60% of total volatiles). Their GC–MS profiling indicates that sabinene (27.86%) and terpinen-4-ol (13.63%) are the major constituents. This chemical distribution correlated with potent cytotoxic efficacy, as ethanol and methanol extracts achieved greater than 80% inhibition of prostate (DU-145, PC-3) and uterine (SK-UT-1) cancer cell line viability, yielding IC_50_ values as low as 1.7 µg/mL. This study supports the traditional use of E. floribundus in several South African communities [Contribution 3].

Guetteche et al. present the characterization of the phenolic profiles of hydroethanolic (EAEE) and ethyl acetate (EAAE) extracts of *Echium asperrimum* and the evaluation of their antibacterial, antioxidant, anti-cholinesterase, and antiproliferative activities. LC–ESI–MS/MS analysis indicates that *p*-coumaric acid, vanillic acid, hydroxybenzaldehyde, and gentisic acid are the major phenolic components of EAAE, whereas *p*-coumaric acid, salicylic acid, sinapic acid, and trans-ferulic acid are predominant in EAEE. Furthermore, EAAE exhibits a dose-dependent antiproliferative activity at concentrations of 50 and 100 µg/mL, with an IC50 value of 83.09 ± 6.50 µg/mL. However, further studies are needed to clarify the contribution of individual compounds and to define the molecular mechanism of action [Contribution 4].

Plazas et al. present a review on the use of molecules isolated from tropical American plants as anti-inflammatory agents and highlight their potential in cytokine storm management (a critical factor in severe inflammatory states such as COVID-19). Information concerning the more important groups of metabolites that serve as the basis for new anti-inflammatory drug design is presented; this review highlights how flavonoids (quercetin, luteolin, and epigallocatechin-3-gallate), polyphenols (resveratrol, curcumin, and rosmarinic acid), alkaloids (berberine, harmine, and mitraphylline), and terpenoids (geraniol, curcumol, parthenolide, and ursolic acid) modulate immune pathways through the downregulation of NF-κB activity and the inhibition of the release of proinflammatory mediators, including TNF-α, IL-6, and IL-1β, in vitro [Contribution 5]. Similarly, Mosquera-Morales et al. present a comprehensive systematic review of the in vitro antiproliferative activity in plants of the genus *Tabebuia*. The most studied species are *T. avellanedae*, *Tabebuia impetiginosa*, *Tabebuia rosea*, and *Tabebuia chrysantha* (*Handroanthus chrysanthus*). Several molecules have been isolated from these plants, the most studied of which is β-lapachone (a naphthoquinone); however, molecules with antiproliferative potential belonging to groups such as iridoids, flavonoids, quinones, furanonaphthoquinones, triterpenes, and polysaccharides have also been isolated and reported [3,4]. Several synthetic derivatives structurally related to β-lapachone, α-lapachone, and 2-acetylfuronaphtoquinone have also been evaluated in vitro against different cancer cell lines. Naphthoquinone modifications and related compounds derived from *Tabebuia* can increase antiproliferative activity, allowing the development of new anticancer agents [Contribution 6]. These two reviews provide key information to validate the use of isolated or modified molecules in preclinical and clinical studies.

Moldoch et al. analyze the utility of monoterpene-based molecular scaffolds—such as carvacrol, carvone, citral, menthol, menthone, β-pinene, thymol, and verbenone—as a “natural toolbox” for modern drug development. The newly designed derivatives exhibit various biological activities, including anticancer, antibacterial, antiviral, and neuroprotective effects. The authors explain how these skeletons can be synthetically modified to increase bioavailability and biological activity [Contribution 7].

The studies presented in this Special Issue demonstrate that natural products remain important resources for drug discovery when they are approached systematically. By refining the structural scaffolds provided by nature and elucidating the precise molecular mechanisms underlying their biological activity, we can develop the next generation of safer, more effective therapeutic agents to treat the global burden of inflammatory and oncological diseases. Also, the contributions reinforce the considerable pharmacological promise of natural products while also highlighting the challenges that remain before these findings can be translated into therapeutic applications. In vitro antioxidant, anti-inflammatory, and anticancer assays provide valuable mechanistic and screening information, but they should be regarded as early-stage evidence rather than definitive evidence of therapeutic efficacy. Future investigations should place greater emphasis on pharmacokinetic and pharmacodynamic characterization, since poor solubility, limited absorption, extensive metabolism, and rapid elimination may substantially restrict the bioavailability of otherwise potent natural compounds. Standardization and quantitative characterization of extracts and their active constituents are likewise essential to ensure reproducibility and establish meaningful dose–response relationships. Importantly, promising in vitro findings should be systematically advanced into well-designed animal studies that establish efficacy, tissue exposure, safety, and mechanisms in physiologically relevant settings. Finally, carefully designed clinical trials will be necessary to determine whether the promising antioxidant, anti-inflammatory, and anticancer activities identified in preclinical studies translate into clinical practice. Closing this translational gap will be critical for converting the remarkable chemical and biological diversity of natural products into safe, reproducible, and evidence-based therapeutic interventions.
